# A Novel Method for Stabilizing Zein Gel Particles to Salt Ion-Induced Aggregation

**DOI:** 10.3390/molecules26051458

**Published:** 2021-03-08

**Authors:** Yiquan Zhang, Jiaqiang Huang, Fazheng Ren, Yi Li, Yi Tong, Pengcheng Wen, Pengjie Wang

**Affiliations:** 1 College of Food Science and Engineering, Gansu Agricultural University, Lanzhou 730070, China; zhangyiquan111@163.com; 2 Department of Nutrition and Health, China Agricultural University, Beijing 100083, China; bornhuang@foxmail.com (J.H.); renfazheng@cau.edu.cn (F.R.); 3 Jilin COFCO Biochemistry Co., Ltd., Changchun 130033, China; liyi@cofco.com (Y.L.); tongyi@cofco.com (Y.T.)

**Keywords:** zein, tannic acid, complex gel particles, ionic strength, stability

## Abstract

The destabilization of zein gel particles by salt ions seriously limits their practical application. In this study, zein gel particles exhibiting excellent stability to salt ions were developed by grafting gum arabic with tannic acid. Gum arabic (GA) was first coated onto the surface of zein gel particles, followed by addition of tannic acid to further reinforce non-covalent cross-linking between GA and the zein gel particle surface. The stability of the gel particle dispersions was characterized by Turbiscan analysis, gel particle diameter changes and visual inspection of phase separation. The tannic acid-treated zein–GA gel particles were highly protected from precipitation or aggregation in the presence of NaCl (0–3 mol/L) at different pH values (4.0, 7.0 or 8.5). The gel particles prepared in this study will therefore have broader applicability in different pH and salt ions ion environments.

## 1. Introduction

Zein is a water-insoluble plant macromolecule that accounts for 50% or more of proteins in the corn endosperm [1]. Based on structure and solubility, it can be divided into α-zein, β-zein, γ-zein and δ-zein [2]. Due to its excellent self-assembly properties, good biocompatibility and degradability, zein can be used to prepare edible gel particles [3]. In recent years, zein gel particles have been widely used as carriers for hydrophobic nutrients, including curcumin [4], beta-carotene [5], resveratrol [6], and tocopherol [7], especially in fat-free or fat-reduced food systems. After being loaded into the zein particles, the stability of nutrients to heat resistance, light fastness and better oxidation could be greatly enhanced.

However, zein contains a large proportion of hydrophobic amino acids, which facilitate aggregation through their hydrophobic surfaces. This can lead to precipitation of zein gel particles and greatly restricts their application [8]. To overcome this limitation, many studies have explored complexation of zein with hydrophilic polymers through non-covalent interactions. These polymers include gum arabic (GA) [9], pectin [10], chitosan [11] and propylene glycol alginate [12]. It has been shown that the complex gel particles are more resistant to aggregation over a wide pH range [9]. The enhanced stability of the complex particles can be reflected in the particle-sized diameters (<400 nm), polydispersity index (<0.4) and zeta–potentials (absolute value >20 mV) [13,14,15,16].

In practice, however, the complex gel particles are often quite sensitive to salt ions in the microenvironment [13]. Even at quite low salt ions concentrations (20 mmol/L NaCl, for example), the complex gel particles readily aggregate and precipitate, which is because the electrostatic interactions between zein and stabilizing polymers can be weakened by ions, leading to desorption of the stabilizing polymers from the zein gel particles [17]. Therefore, how to inhibit the desorption of polymers from zein gel particles in ionic environments should be considered.

GA molecules can be adsorbed onto the surface of zein gel particles through non-covalent interactions, forming a core-shell structure with zein as the core and GA as the shell [9,14]. When pH > pI, most residues in proteins are negatively charged, but some parts or fragments remain positively charged due to the presence of basic amino acid residues, which could interact with the negatively charged GA [18], forming complex gel particles with core-shell structure [19].

**Figure 1:**
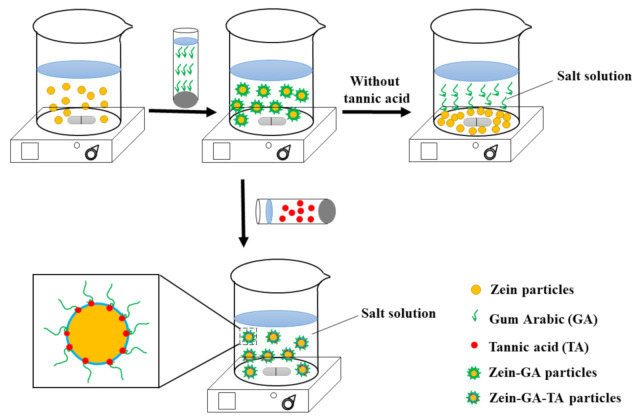
The preparation diagram of the zein–gum -arabic–tannic acid (-zein-GA-TA) gel particles.

Tannic acid (TA) contains a large number of hydroxyl groups (~25) that can interact with carbonyl groups on proteins [20]. These interactions could facilitate the formation of relatively stable hydrogen bonds [21,22], generating cross-linkage between different protein domains (as shown in Figure 1). The introduction of numerous hydrophilic hydroxyl groups on the surface might stabilize the shell-core structural gel particles in high ionic strength microenvironments.

Herein, we propose a new method for the preparation of gel particles that are stable to aggregation in microenvironments of different ionic strength. The method of preparation is shown in Figure 1. Gum arabic is a well-known, naturally occurring protein–polysaccharide conjugate, and the isoelectric point (pI) of zein is 6.2 [23]. Gum arabic could adsorb onto the surface of zein gel particles through electrostatic or hydrophobic interactions [9]. The introduction of tannic acid could cross-link the protein part of GA with protein on the surface of the zein gel particle via hydrogen bonds. The presence of polysaccharide groups contributed by the gum arabic enables generation of a highly hydrophilic layer at the surface of the complex gel particles. These newly formed gel particles might be stable to different ionic strengths. The main purpose of this study was to examine this method, particularly with regard to the stability of the gel particles to different ionic strengths.

## 2. Results and Discussion

### 2.1. Appearance of the Dispersions

**Figure 2:**
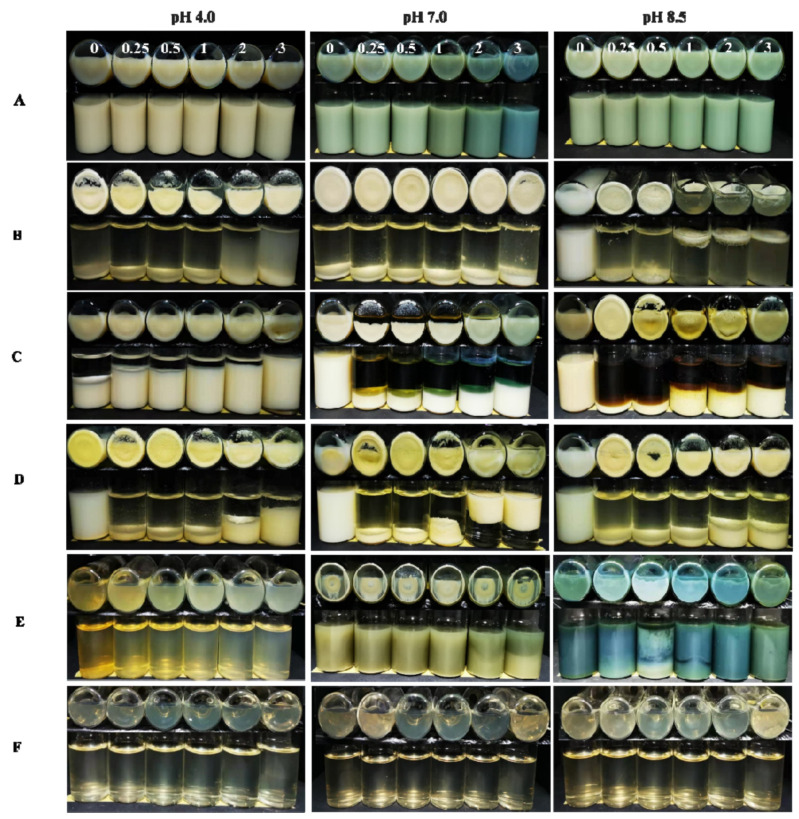
The appearance of dispersions including zein–GA–TA (**A**), zein–GA (**B**), zein–TA (**C**), ZP (**D**), GA+TA (**E**) and GA (**F**) as a function of NaCl concentration (0, 0.25, 0.5 1, 2, 3 mol/L) at pH 4.0, 7.0, or 8.5.

The appearances of the gel particle dispersions at different NaCl concentrations and pH values after centrifugation are shown in Figure 2. When the pH was 8.5, in the absence of NaCl, no macroscopic gel particles or sedimentation were observed in any of the dispersions; at NaCl concentrations of 0.25, 0.5, 1, 2 and 3 mol/L, visual sedimentation was observed in dispersions containing zein or zein–GA. However, for the zein–GA–TA dispersions, no visual sedimentation was observed. This indicated that TA efficiently stabilized the zein–GA complex gel particles to aggregation in the presence of NaCl.

At pH 7.0, in the absence of NaCl, sedimentation was observed in the zein and zein–GA dispersions. This is likely because pH 7.0 is quite close to the pI of zein; the net charge on the surface of zein gel particles is limited, while surface hydrophobicity is greater than at other pH values. Zein gel particles easily aggregate through hydrophobic interactions. However, no visual sedimentation was observed in the zein–GA–TA dispersions. This suggests that TA efficiently stabilized the zein–GA complex gel particles to aggregation in the presence of NaCl. Similar results were also found at pH 4.0.

### 2.2. Backscattering Changes

**Figure 3:**
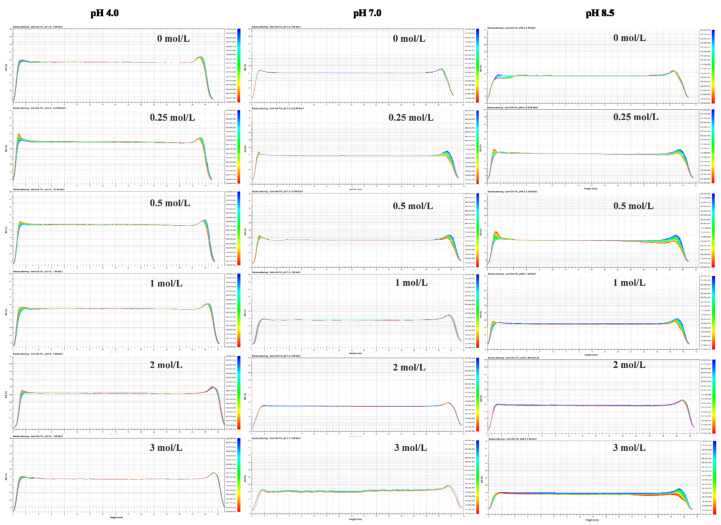
Backscattering changes of zein–GA–TA gel particle dispersions at different pH values (4.0, 7.0, or 8.5) and different concentrations (0, 0.25, 0.5 1, 2, 3 mol/L) of sodium chloride. And x-axis means height (mm), y-axis means BS (%).

The physical destabilization process of colloids is easily detected from their optical properties. When the destabilization process involves sedimentation, the backscattering intensity (BS) of the gel particle dispersions increases at the bottom of the test tube while decreasing at the top of the tube. When the destabilization process involves aggregation, the BS of the gel particle dispersions increases at the middle of the test tube. As shown in Figure 3, there was little variation of BS in the middle or bottom parts of the zein–GA–TA dispersions containing 0, 0.25, 0.5, 1, 2 and 3 mol/L NaCl at pH values of 8.5, 7.0 or 4.0. However, the BS of the other groups (zein–GA, zein–TA, ZP dispersions) showed big changes over time (Supplementary Material 1, Figures S1–S5). This indicated that the TA-treated zein–GA gel particles showed excellent stability in the presence of salt ions (Supplementary Material 2).

### 2.3. Gel Particle Size, Polydispersity Index, and Zeta–Potential

**Figure 4:**
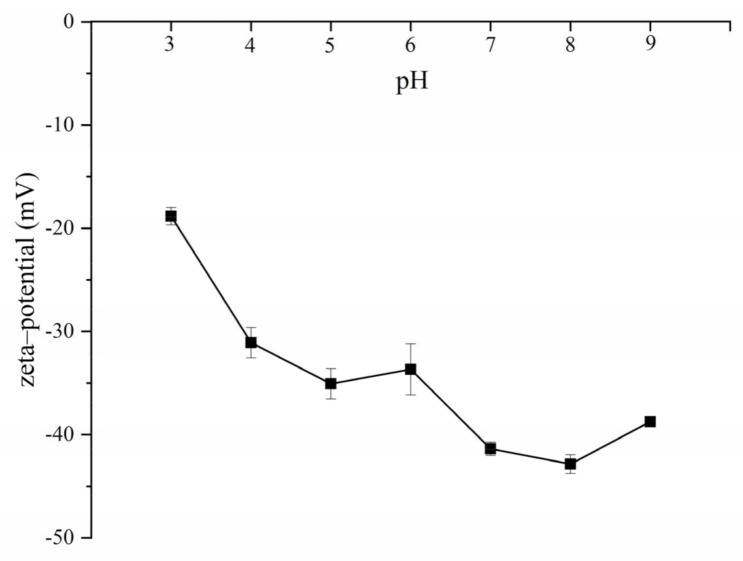
Influence of pH on the zeta–potential of zein–GA–TA gel particle dispersions.

The diameters and polydispersity index (PDI) of gel particles after 30 days storage are shown in Table 1. When the pH was 8.5, in the absence of NaCl, the diameters of zein, zein–GA, and zein–TA were 88.0 ± 4.1, 122.2 ± 1.2, and 77.9 ± 2.4 nm, respectively. This indicated that the addition of GA increased the size of zein gel particles, while addition of TA had the opposite effect. This might be because GA formed a shell on the zein gel particle surfaces, while the decrease of zein-TA average gel particle size is due to the higher surface charge density [15]. When different amounts of sodium chloride are added into ZP, zein–GA, or zein–TA dispersions, they all aggregate and precipitate. However, after TA processing, addition of sodium chloride to the zein–GA–TA gel particle dispersions did not cause precipitation. The gel particle size of zein–GA–TA dispersions increased (from 155.0 ± 3.0 to 247.8 ± 8.7 nm) at first and then decreased (to 196.0 ± 10.5 nm) as the sodium chloride concentration was increased (from 0 to 3 mol/L). The increase in gel particle size may be due to electrostatic shielding at low concentrations of sodium chloride [13,17,24]. However, when the sodium chloride concentration was greater than 0.25 mol/L, the gel particle size gradually decreased. It may be that the high concentration of salt ions cause the gel particles to shrink. The morphology of zein–GA–TA gel particles (in the absence of sodium chloride) was measured as spherical by scanning electron microscopy (SEM) (Figure S6). The diameter of the zein–GA–TA particles measured by SEM (142.6 ± 19.8, 125.8 ± 16.2 and 135.7 ± 19.4 nm respectively at pH 4.0, 7.0 and 8.5) was smaller than that determined by light scattering. Dynamic light scattering basically measures the hydrodynamic diameter of the particles, while SEM required the samples to be in dehydrated state, leading to the shrinkage of gel particles. When the pH value was 8.5, the PDI of zein–GA–TA gel particle dispersions under different sodium chloride concentrations was about 0.32 or less. Similar results were also found at pH 4.0 and 7.0. The influence of pH on the zeta–potential of zein–GA–TA gel particle dispersions is shown in Figure 4. It can be observed the zeta–potential of zein–GA–TA gel particles were all negatively charged from 3 to 9.

## 3. Materials and Methods

### 3.1. Materials

Zein, gum arabic (GA) and tannic acid (TA) were purchased from Sigma-Aldrich Corporation (St. Louis, MO, USA). Other chemical agents such as ethanol, sodium chloride (NaCl), hydrochloric acid (HCl) and sodium hydroxide (NaOH) were obtained from Yongda Chemical Reagent Corporation, Ltd. (Tianjin, China). The water used in this study was tap water purified to 18.2 MΩ using an Arium Comfort Ⅱ purifier (Sartorius Corporation, Göttingen, Germany).

### 3.2. Preparation of Gel Particles

Zein powder (5 g) was added to 80% (*v/v*) ethanol/water (100 mL) with continuous stirring at room temperature (~25 °C) for 1 h. The pH of the solution was adjusted to 9.0 with 0.1 mol/L NaOH solution. GA (10 g) was added to ultrapure water (240 mL, 50 °C) and stirred at a speed of 600 rpm for 1 h to prepare a GA dispersion. The pH was maintained at 9.0 with 0.1 mol/L NaOH solution and the dispersion was cooled to room temperature for later use.

**Figure 5:**
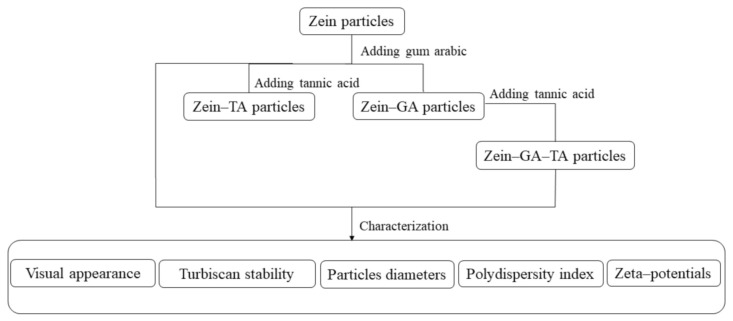
The preparation procedure of particles in this study.

Gel particles were fabricated using an anti-solvent precipitation method with some modifications [9]. The zein aqueous ethanol solution (20 mL, the dropping rate is 10 mL/min) was added dropwise to ultrapure water (100 mL) to prepare a zein gel particle dispersion. The dispersion was stirred at 600 rpm for 1 h and the pH was kept at 9.0 with 0.1 mol/L NaOH solution. The GA dispersion (the dropping rate is 3 mL/min) was then added into the zein gel particle dispersion at a zein–GA mass ratio of 1:2 with magnetic stirring at 600 rpm for 1 h. The obtained zein–GA complex gel particle dispersion (168 mL) was treated with tannic acid solution (5 mL, 0.04 g/mL) under continuous stirring (600 rpm) at room temperature for 30 min to prepare the zein–GA–TA gel particle dispersion. For storage, 0.04% sodium azide was added in the dispersions to inhibit the microorganism growth. For comparison, different experimental groups were used as follows (Figure 5):

(i) Zein gel particle dispersion (ZP). Zein powder (5 g) was added to 80% (*v/v*) ethanol/water (100 mL) with continuous stirring at room temperature (~25 °C) for 1 h. The pH of the solution was adjusted to 9.0 with 0.1 mol/L NaOH solution. The zein aqueous ethanol solution (20 mL, the dropping rate is 10 mL/min) was added to ultrapure water (100 mL) to prepare a zein gel particle dispersion. The dispersion was stirred at 600 rpm for 1 h and the pH was kept at 9.0 with 0.1 mol/L NaOH solution.
(ii) GA dispersion. GA powder (10 g) was added to ultrapure water (240 mL, 50 °C) and stirred at 600 rpm for 1 h to prepare a GA dispersion. The pH was maintained at 9.0 with 0.1 mol/L NaOH solution and the dispersion was cooled to room temperature for further use.
(iii) Zein–GA gel particle dispersion. Zein powder (5 g) was added to 80% *(v/v*) ethanol/water (100 mL) with continuous stirring at room temperature (~25 °C) for 1 h. The pH of the solution was adjusted to 9.0 with 0.1 mol/L NaOH solution. The zein aqueous ethanol solution (20 mL) was added dropwise to ultrapure water (100 mL) to prepare a zein gel particle dispersion. The dispersion was stirred at 600 rpm for 1 h and the pH was kept at 9.0 with 0.1 mol/L NaOH solution. GA powder (10 g) was added to ultrapure water (240 mL, 50 °C) and stirred at 600 rpm for 1 h to prepare a GA dispersion. The pH was maintained at 9.0 with 0.1 mol/L NaOH solution and the dispersion was cooled to room temperature for later use. The GA dispersion was then added dropwise into the zein gel particle dispersion at a zein:GA mass ratio of 1:2 with magnetic stirring at 600 rpm for 1 h to obtain the zein–GA complex gel particle dispersion.
(iv) Zein–TA gel particle dispersion. Zein powder (5 g) was added to 80% (*v/v*) ethanol/water (100 mL) with continuous stirring at room temperature (~25 °C) for 1 h. The pH of the solution was adjusted to 9.0 with 0.1 mol/L NaOH solution. The zein aqueous ethanol solution (20 mL) was added dropwise to ultrapure water (100 mL) to prepare a zein gel particle dispersion. The dispersion was stirred at 600 rpm for 1 h and the pH was kept at 9.0 with 0.1 mol/L NaOH solution. Tannic acid solution (5 mL, 0.04 g/mL) was then added to the zein gel particle dispersion (120 mL) while stirring (600 rpm) at room temperature for 30 min.
(v) GA+TA dispersion. GA powder (10 g) was added to ultrapure water (240 mL, 50 °C) and stirred at 600 rpm for 1 h to prepare a GA dispersion. The pH was maintained at 9.0 with 0.1 mol/L NaOH solution and the dispersion was cooled to room temperature for later use. Tannic acid solution (5 mL, 0.04 g/mL) was added to the GA dispersion (48 mL) at a GA:TA mass ratio of 10:1 and stirred (600 rpm) at room temperature for 30 min.

### 3.3. Characterization of Gel Particle Stability to Salt Ions

#### 3.3.1. Visual Appearance

Different amounts of NaCl were added into each group to obtain various concentrations (0, 0.25, 0.5, 1, 2 and 3 mol/L), after which the pH was adjusted to 4.0, 7.0, or 8.5, respectively. Samples of the dispersions (12 mL) were added to 15 mL centrifuge tubes followed by centrifugation at 4500× *g* for 20 min at 25 °C.

#### 3.3.2. Turbiscan Stability

Turbiscan (Formulaction, L’Union, France) was used to detect and quantify the destabilization mechanisms of the gel particle dispersions [25]. A pulsed near-infrared light source is combined with two synchronous detectors in the detection head, which moves along a cylindrical unit to record data from backscattering and transmission intensity. Different amounts of NaCl were added into each group to obtain various concentrations (0, 0.25, 0.5, 1, 2 and 3 mol/L), after which the pH was adjusted to 4.0, 7.0, or 8.5, respectively. Measurements were carried out every 10 min at 25 °C for 24 h.

#### 3.3.3. Gel Particle Size, Polydispersity Index, Zeta–Potential and Morphology

After stored at room temperature (~25 °C) for 30 days, the gel particle dispersions (100 μL) were diluted 200 times with sodium chloride solutions at different concentrations, with the same pH and NaCl concentrations as the original gel particle dispersions, for gel particle size and polydispersity index measurements using a Nano Zetasizer (Malvern, Worcestershire, UK) [26]. The solvent refractive indices used for different concentrations of NaCl (0, 0.25, 0.5, 1, 2 and 3 mol/L) were 1.33, 1.33, 1.34, 1.34, 1.35 and 1.36, respectively, while the corresponding solvent viscosities used were 0.89, 0.91, 0.93, 0.98, 1.10 and 1.25 cP, respectively. All measurements were performed at 25 °C. Measurements were made after 120 s of equilibration to avoid multiple scattering effects.

The zeta–potential of zein–GA–TA gel particle dispersions was measured using a Nano Zetasizer (Malvern, Worcestershire, UK) The samples were diluted 200 times with pure water.

The morphology of zein–GA–TA gel particles (in the absence of sodium chloride) was measured by scanning electron microscopy (SU8010, Hitachi, Tokyo, Japan) at an accelerating voltage of 5.0 kV. Before observation, the surfaces of samples were sprayed with gold to avoid charging under the electron beam.

#### 3.3.4. Statistical Analysis

All experiments were repeated in triplicate. SPSS Statistics 24 was used for statistical analysis. Differences between the data were assessed using one-way ANOVA and Duncan’s test; *p* < 0.05 was considered significant.

## 4. Conclusions

Stabilizing gel particles to salt ion induced aggregation is important for their practical applications. The preparation of zein–GA–TA gel particles proposed in this paper is simple and effective. Compared with zein–polysaccharide complex gel particles assembled only by electrostatic or hydrophobic interactions, the zein–GA–TA gel particles in this study did not precipitate or aggregate in different salt ions concentrations (0–3 mol/L) at various pH values (4.0, 7.0 or 8.5). The biopolymer gel particles prepared in this study have potential as nanocarriers.

## Figures and Tables

**Table 1:** Mean diameters and polydispersity indices of the gel particles at different pH values.

Sample			Sodium Chloride (mol/L)					
			0	0.25	0.5	1	2	3
pH 4.0	Zein–GA–TA	Diameters (nm)	201.7 ± 9.7 ^c^	268.1 ± 2.8 ^a^	237.7 ± 12.9 ^b^	184.9 ± 6.9 ^d^	185.9 ± 5.4 ^d^	191.2 ± 5.4 ^c,d^
		PDI	0.17 ± 0.01 ^b,c^	0.30 ± 0.08 ^a^	0.20 ± 0.03 ^b^	0.14 ± 0.01 ^b,c^	0.12 ± 0.01 ^c^	0.12 ± 0.01 ^c^
	Zein-GA		*	*	*	*	*	*
	Zein-TA		*	*	*	*	*	*
	ZP		*	*	*	*	*	*
	GA+TA		–	–	–	–	–	–
	GA		–	–	–	–	–	–
pH 7.0	Zein–GA–TA	Diameters (nm)	200.2 ± 13.6 ^b^	240.3 ± 17.8 ^a^	247.1 ± 16.6 ^a^	212.9 ± 10.0 ^b^	202.6 ± 5.1 ^b^	191.2 ± 4.8 ^b^
		PDI	0.21 ± 0.07 ^b^	0.16 ± 0.01 ^b^	0.32 ± 0.08 ^a^	0.18 ± 0.03 ^b^	0.18 ± 0.08 ^b^	0.14 ± 0.00 ^b^
	Zein-GA		*	*	*	*	*	*
	Zein-TA		*	*	*	*	*	*
	ZP		*	*	*	*	*	*
	GA+TA		–	–	–	–	–	–
	GA		–	–	–	–	–	–
pH 8.5	Zein–GA–TA	Diameters (nm)	203.4 ± 9.1 ^b^	247.8 ± 8.7 ^a^	239.5 ± 17.3 ^a^	230.2 ± 4.4 ^a^	210.0 ± 6.9 ^b^	196.0 ± 10.5 ^b^
		PDI	0.19 ± 0.01 ^c^	0.31 ± 0.05 ^a,b^	0.32 ± 0.03 ^a^	0.25 ± 0.01 ^a,b,c^	0.20 ± 0.06 ^c^	0.22 ± 0.09 ^b,c^
	Zein-GA	Diameters (nm)	122.2 ± 1.2	*	*	*	*	*
		PDI	0.16 ± 0.01	*	*	*	*	*
	Zein-TA	Diameters (nm)	77.9 ± 2.4	*	*	*	*	*
		PDI	0.22 ± 0.01	*	*	*	*	*
	ZP	Diameters (nm)	88.0 ± 4.1	*	*	*	*	*
		PDI	0.19 ± 0.01	*	*	*	*	*
	GA+TA		–	–	–	–	–	–
	GA		–	–	–	–	–	–

The values ^a,b,c,d^ are expressed as means ± standard deviation (n = 6); the different alphabets within a row represent significantly different (*p* < 0.05). “*” indicates sample precipitation. “–” indicates no accurate data were available.

## Data Availability

Data is contained within the article.

## Supplementary Materials

The following are available online, Supplementary Material 1, Figure S1: Backscattering changes of zein-GA gel particle dispersions at different pH values (4.0, 7.0, or 8.5) and different concentrations (0, 0.25, 0.5, 1, 2, 3 mol/L) of sodium chloride; Figure S2: Backscattering changes of zein–TA gel particle dispersions at different pH values (4.0, 7.0, or 8.5) and different concentrations (0, 0.25, 0.5, 1, 2, 3 mol/L) of sodium chloride; Figure S3: Backscattering changes of zein gel particle dispersions at different pH values (4.0, 7.0, or 8.5) and different concentrations (0, 0.25, 0.5, 1, 2, 3 mol/L) of sodium chloride; Figure S4: Backscattering changes of GA+TA dispersions at different pH values (4.0, 7.0, or 8.5) and different concentrations (0, 0.25, 0.5, 1, 2, 3 mol/L) of sodium chloride; Figure S5: Backscattering changes of GA dispersions at different pH values (4.0, 7.0, or 8.5) and different concentrations (0, 0.25, 0.5, 1, 2, 3 mol/L) of sodium chloride. Figure S6: SEM image of zein–GA–TA gel particles (in the absence of sodium chloride) at pH 4.0 (A), 7.0 (B), and 8.5 (C); Supplementary material 2, backscattering changes of zein–GA–TA gel particle dispersions at different pH values (4.0, 7.0, or 8.5) and different concentrations (0, 0.25, 0.5 1, 2, 3 mol/L) of sodium chloride.

## Sample Availability

The particles in the study are available from the authors.
