# Supplementary material for: A Novel Method for Stabilizing Zein Gel Particles to Salt Ion-Induced Aggregation
Source: Molecules. 2021 Mar 8;26(5):1458. doi: 10.3390/molecules26051458 (PMC7975981; doi:10.3390/molecules26051458)
Supplement: Supplementary file 1 [file molecules-26-01458-s001.zip › molecules-1102352-supplementary/Supplementary Materals/Supplementary Material 1.docx]

**Fig. S1.**

**
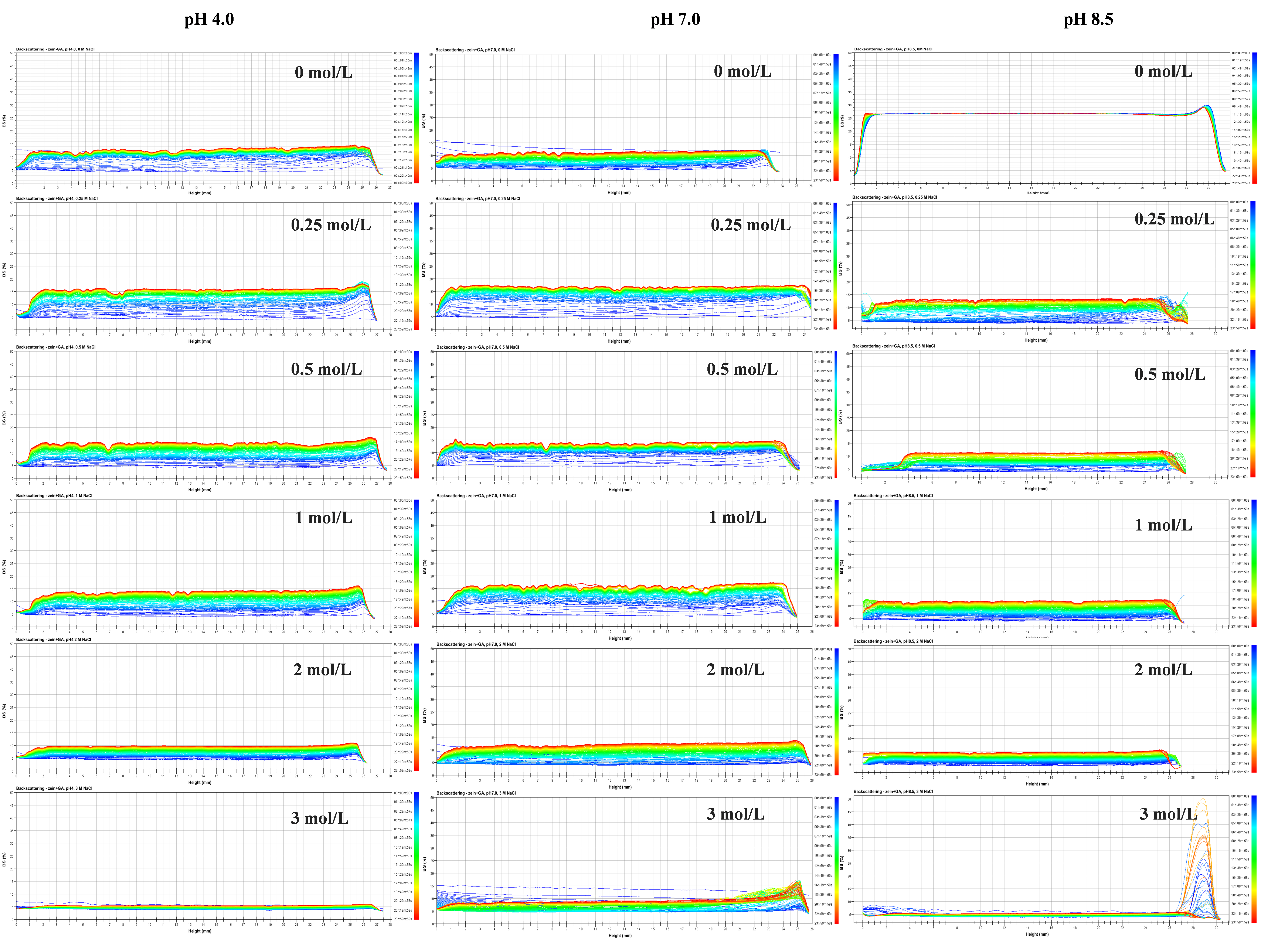
**

**Fig. S1.** Backscattering changes of zein-GA gel particle dispersions at different pH values (4.0,7.0, or 8.5) and different concentrations (0, 0.25, 0.5 1, 2, 3 mol/L) of sodium chloride.

**Fig. S2.**

**
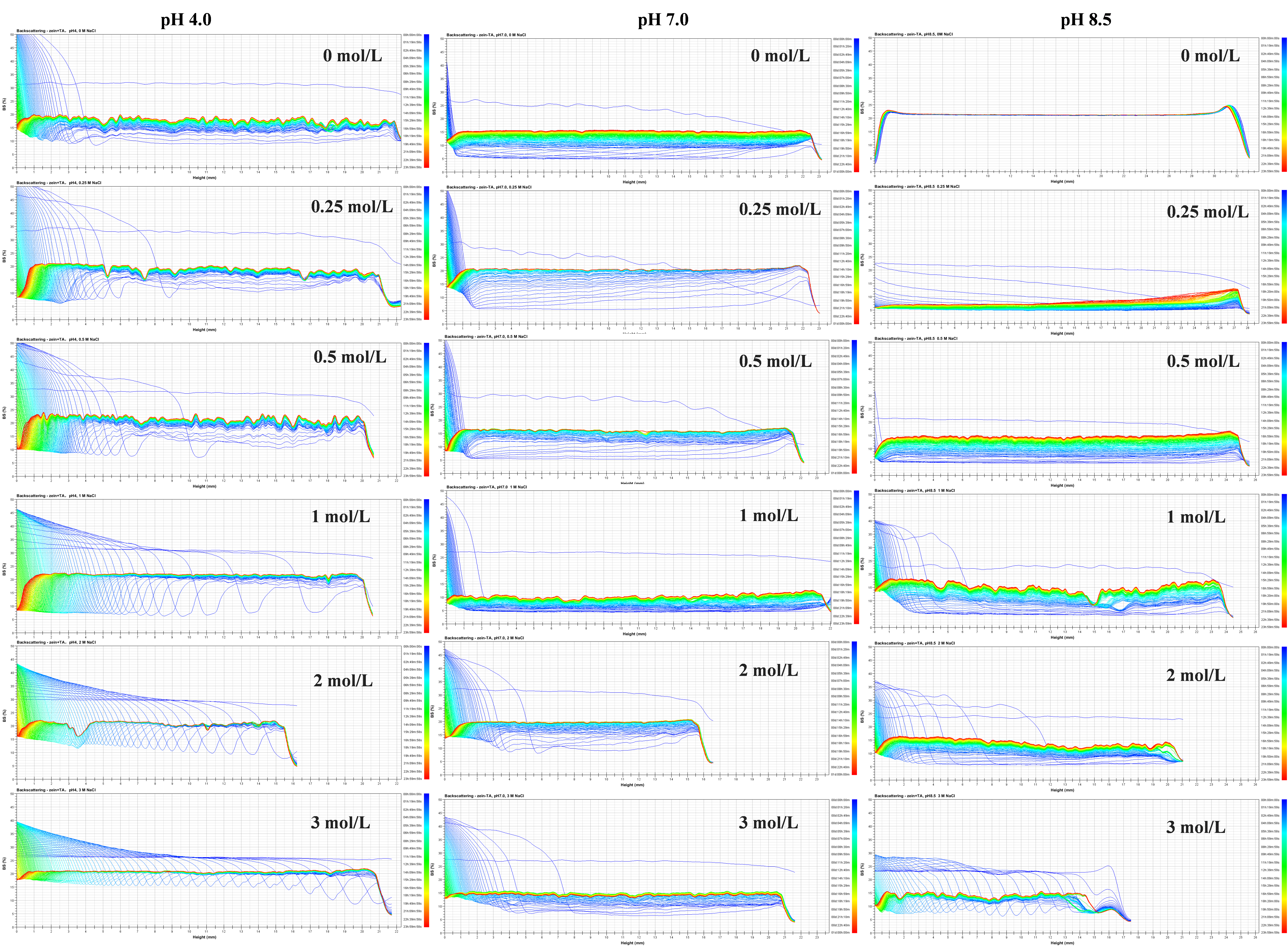
**

**Fig. S2.** Backscattering changes of zein-TA gel particle dispersions at different pH values (4.0,7.0, or 8.5) and different concentrations (0, 0.25, 0.5 1, 2, 3 mol/L) of sodium chloride.

**Fig. S3.**

**
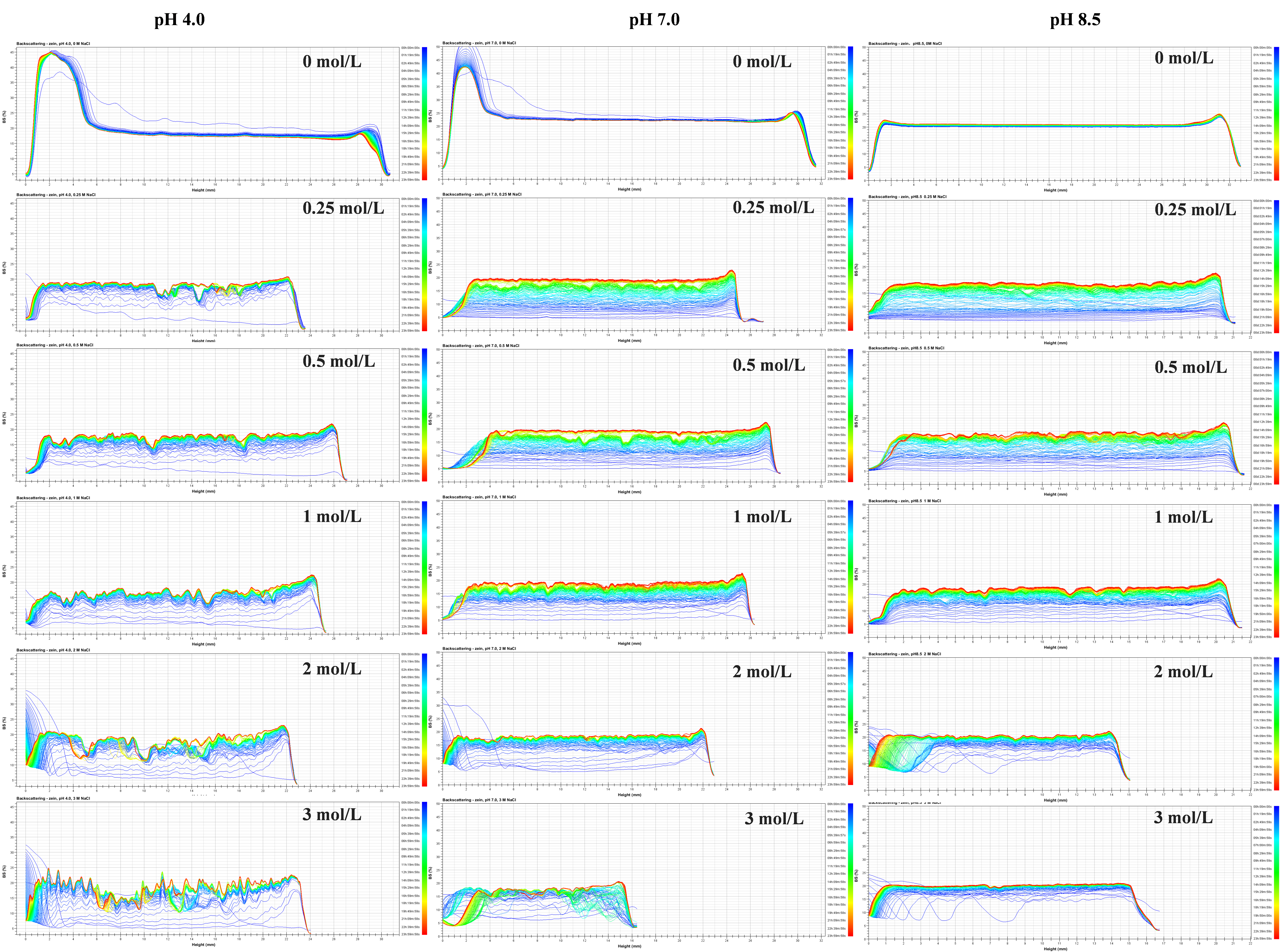
**

**Fig. S3.** Backscattering changes of zein gel particle dispersions at different pH values (4.0,7.0, or 8.5) and different concentrations (0, 0.25, 0.5 1, 2, 3 mol/L) of sodium chloride.

**Fig. S4.**

**
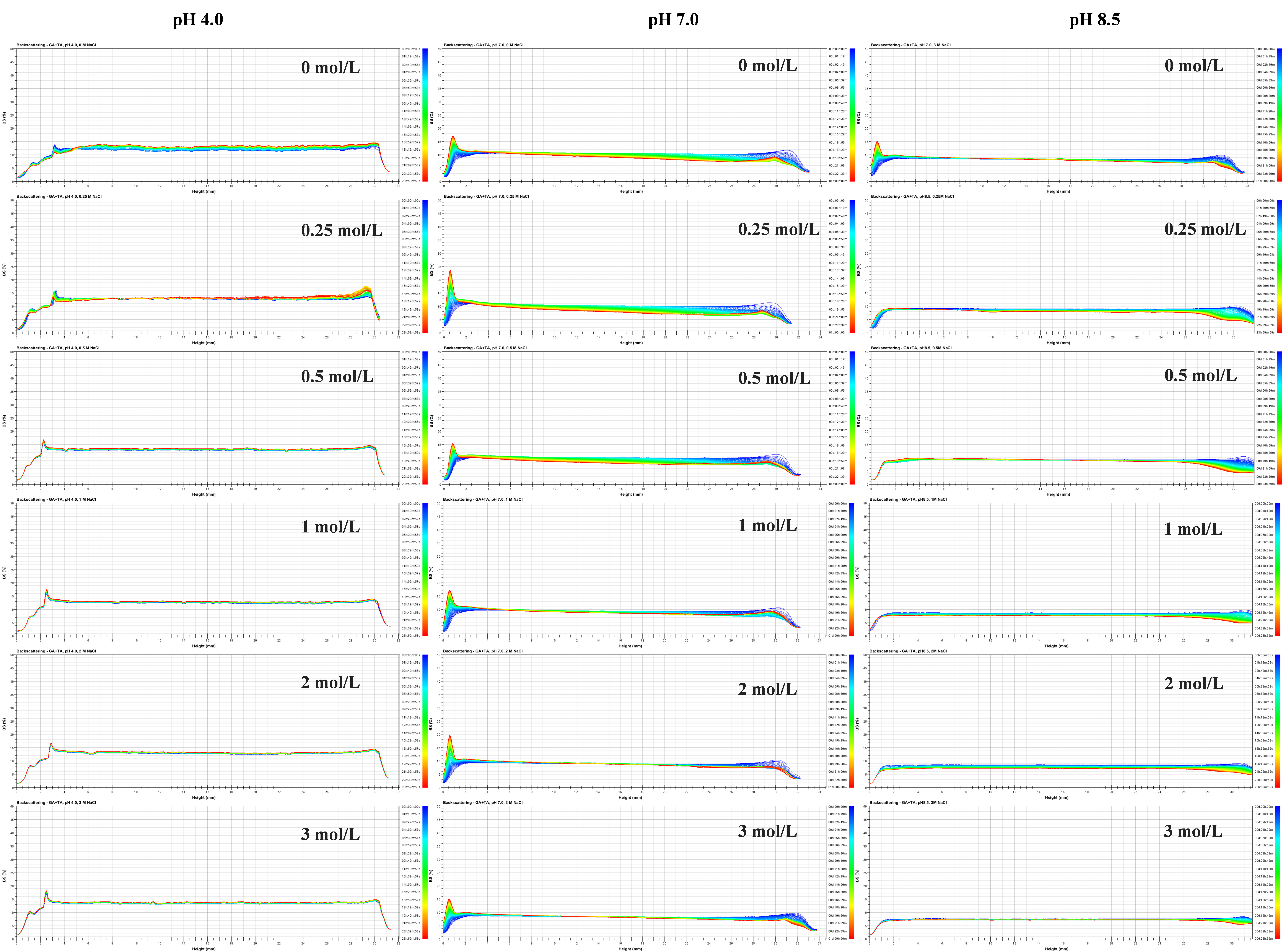
**

**Fig. S4.** Backscattering changes of GA+TA dispersions at different pH values (4.0,7.0, or 8.5) and different concentrations (0, 0.25, 0.5 1, 2, 3 mol/L) of sodium chloride.

**Fig. S5.**

**
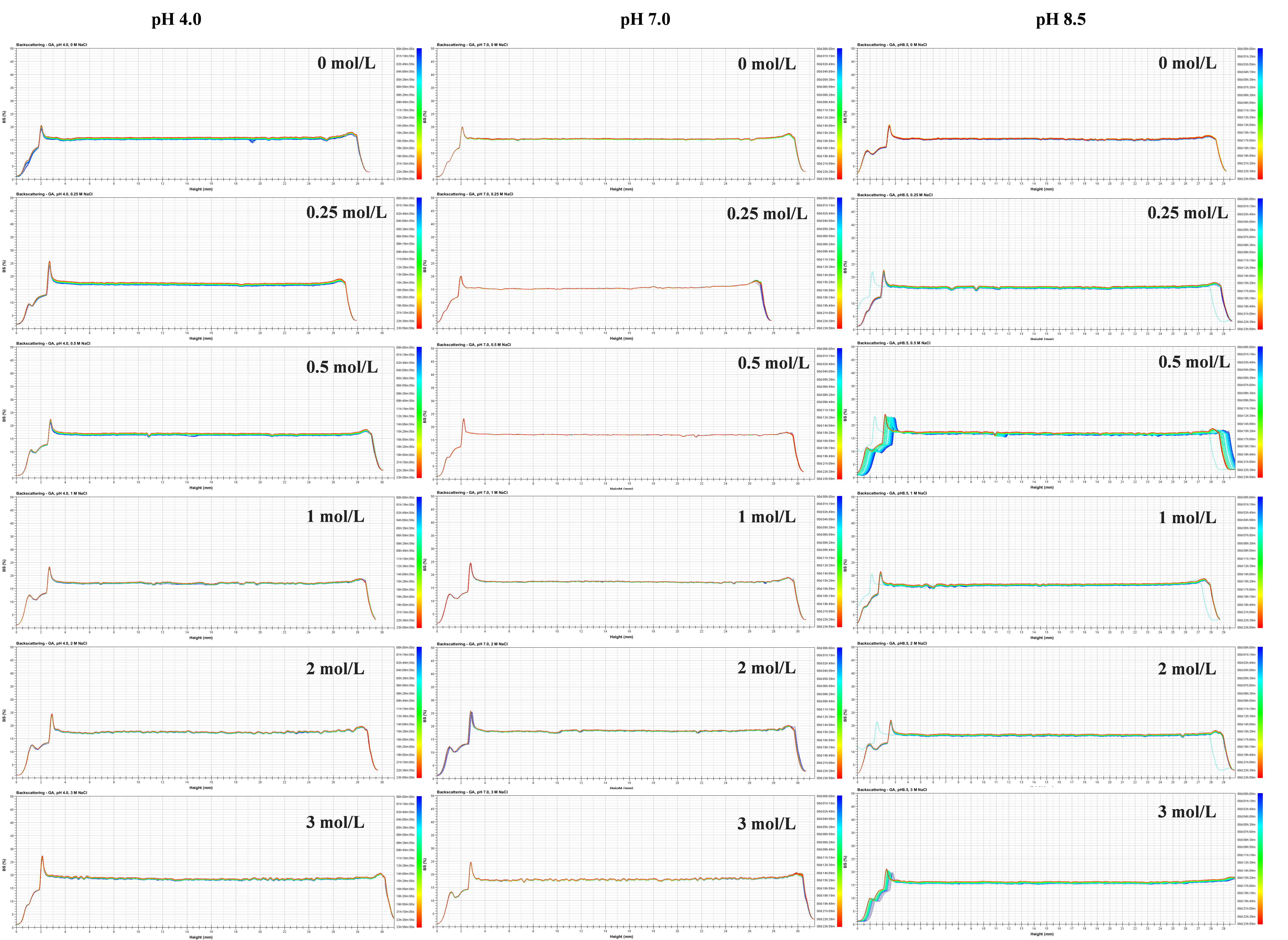
**

**Fig. S5.** Backscattering changes of GA dispersions at different pH values (4.0,7.0, or 8.5) and different concentrations (0, 0.25, 0.5 1, 2, 3 mol/L) of sodium chloride.

**Fig. S6.**


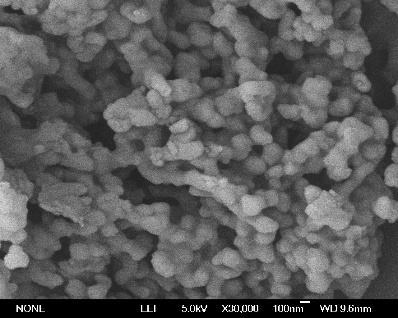

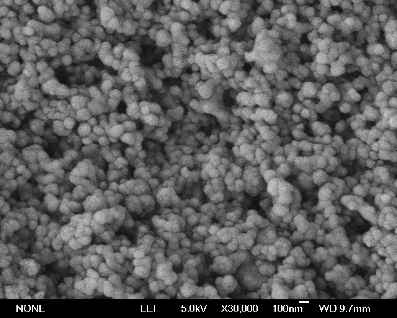

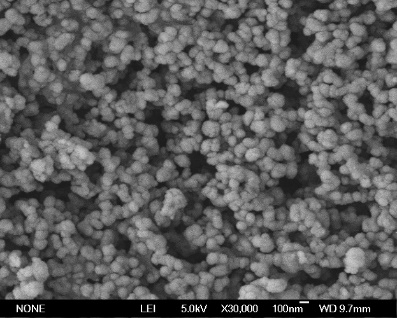


**C**

**B**

**A**

**Fig. S6.** SEM images of zein-GA-TA gel particles (in the absence of sodium chloride) at pH 4.0(A),7.0(B), and 8.5(C).
