# Supplementary material for: A Novel Method for Stabilizing Zein Gel Particles to Salt Ion-Induced Aggregation
Source: Molecules. 2021 Mar 8;26(5):1458. doi: 10.3390/molecules26051458 (PMC7975981; doi:10.3390/molecules26051458)
Supplement: Supplementary file 1 [file molecules-26-01458-s001.zip › molecules-1102352-supplementary/Supplementary Materals/Supplementary Material 2 .docx]

Backscattering changes of zein-GA-TA particle dispersions at different pH values and different concentrations of sodium chloride.

**pH 4.0, 0 mol/L NaCl
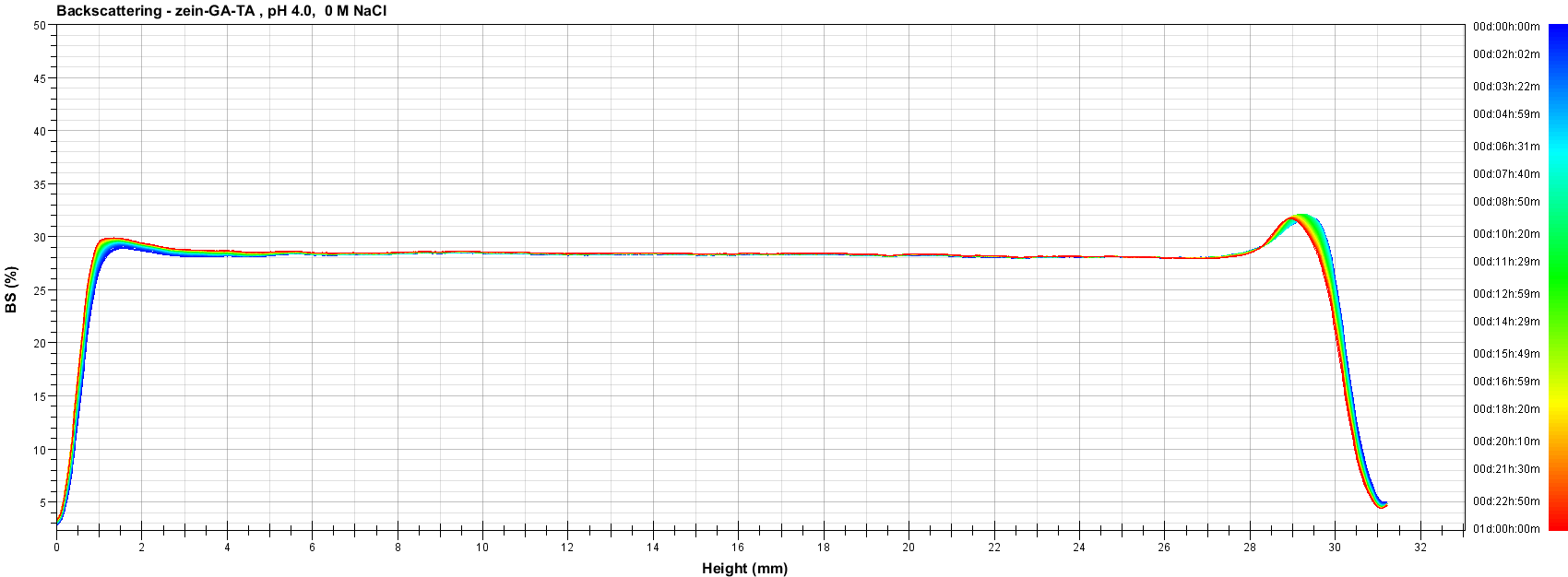
**

**zein-GA-TA**

**pH 4.0, 0.25 mol/L NaCl**

**
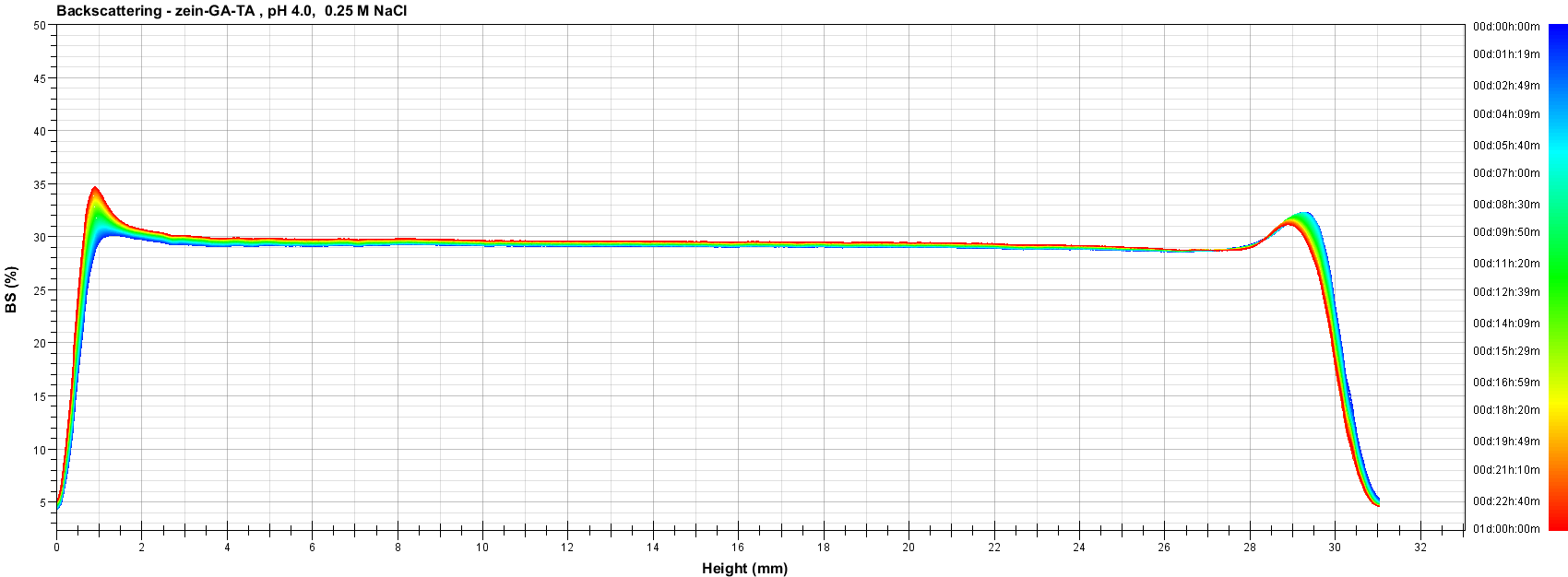
**

**zein-GA-TA**

**pH 4.0, 0.5 mol/L NaCl**


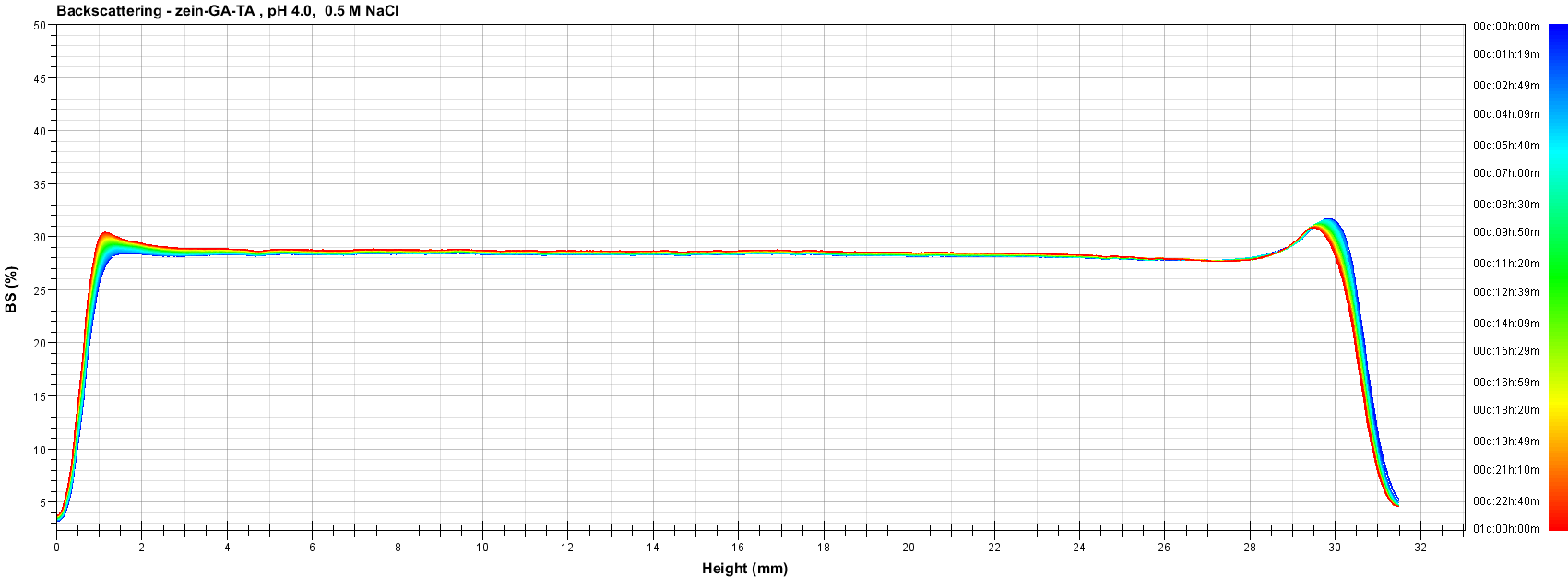


**zein-GA-TA**

**pH 4.0, 1 mol/L NaCl**


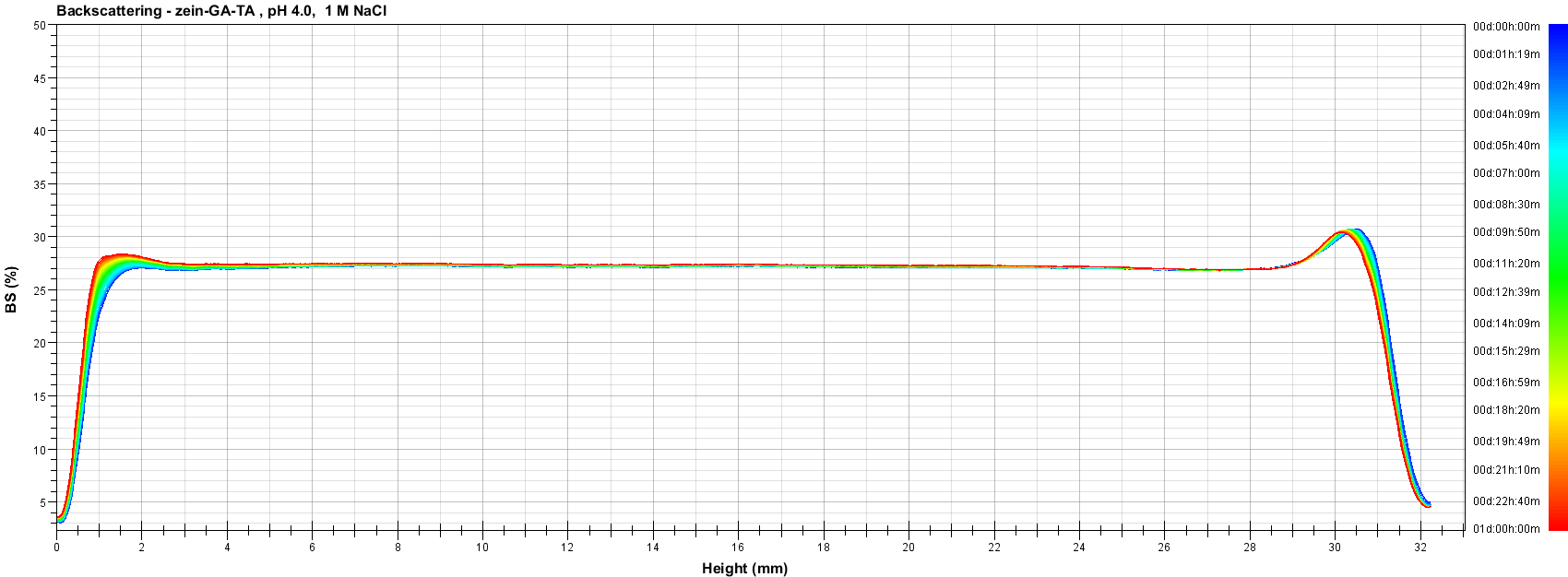


**zein-GA-TA**

**pH 4.0, 2 mol/L NaCl**


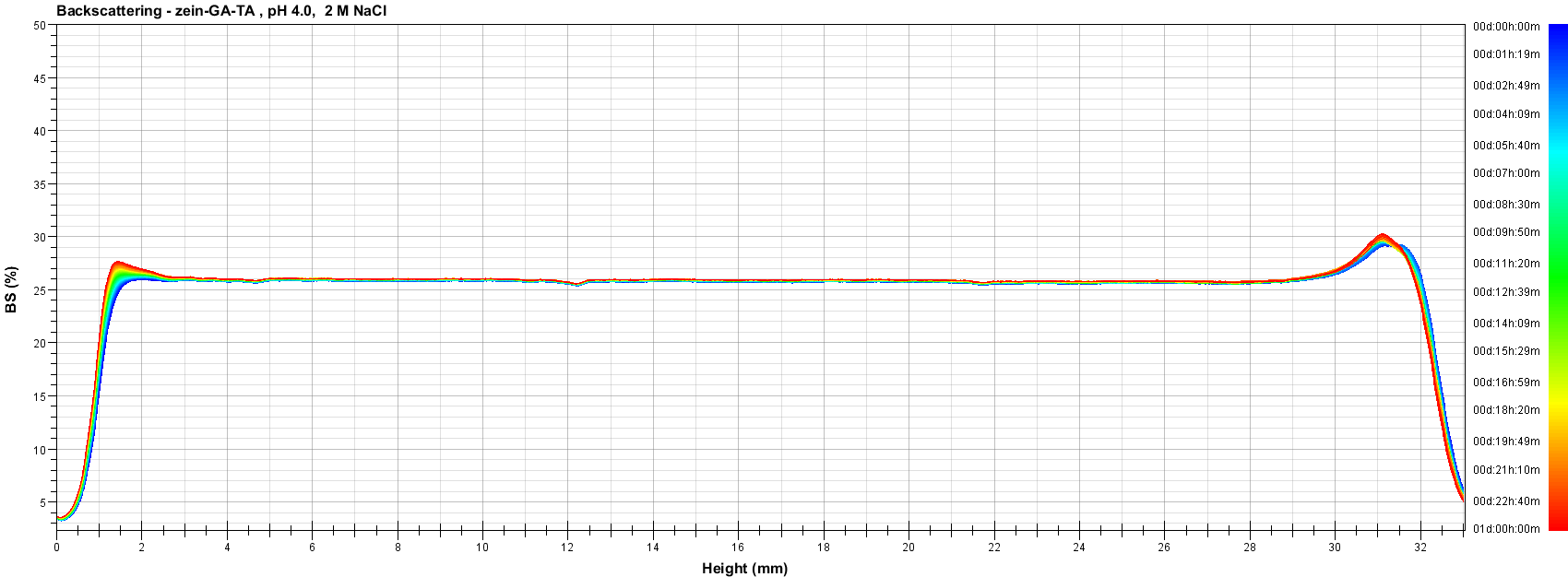


**zein-GA-TA**

**pH 4.0, 3 mol/L NaCl**


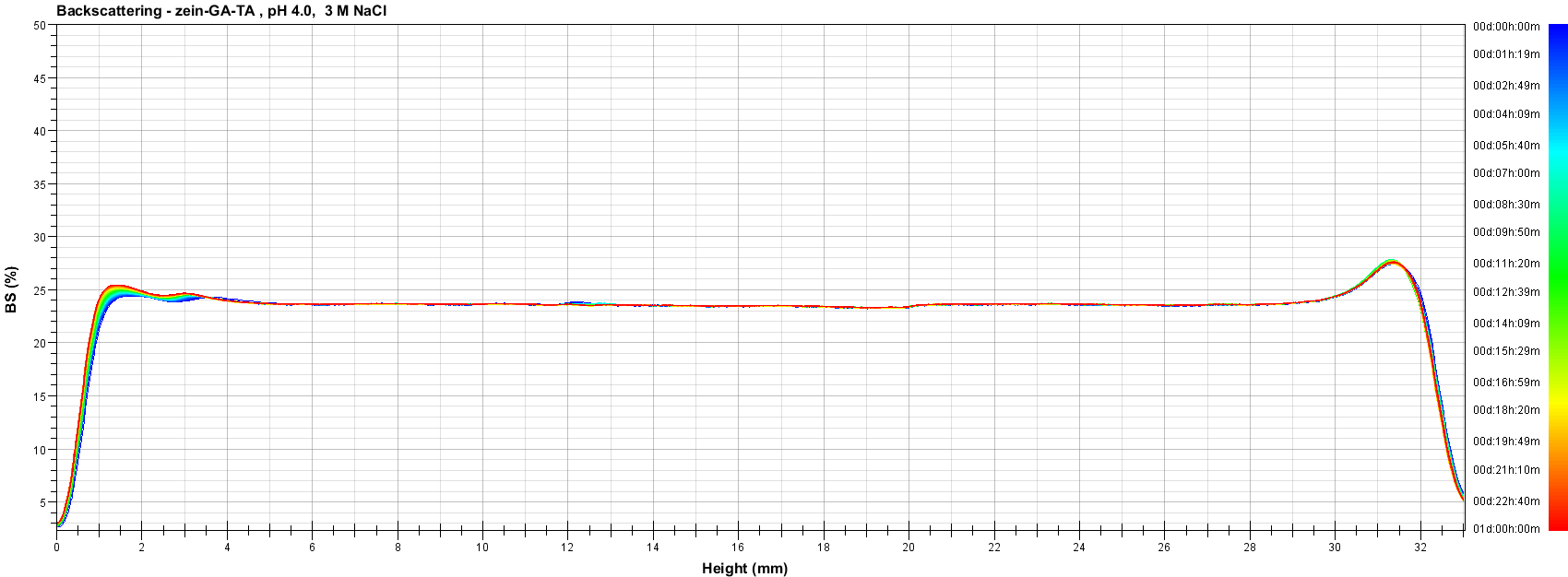


**zein-GA-TA**

**pH 7.0, 0 mol/L NaCl**


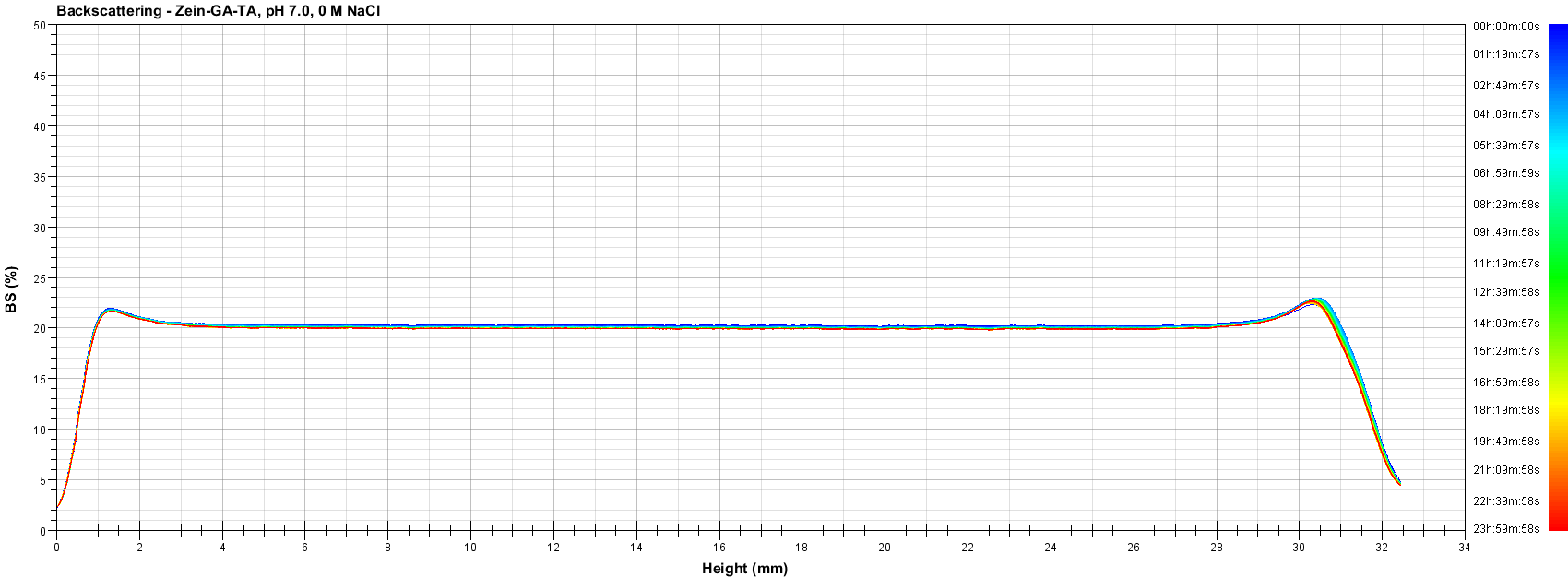


**zein-GA-TA**

**pH 7.0, 0.25 mol/L NaCl**


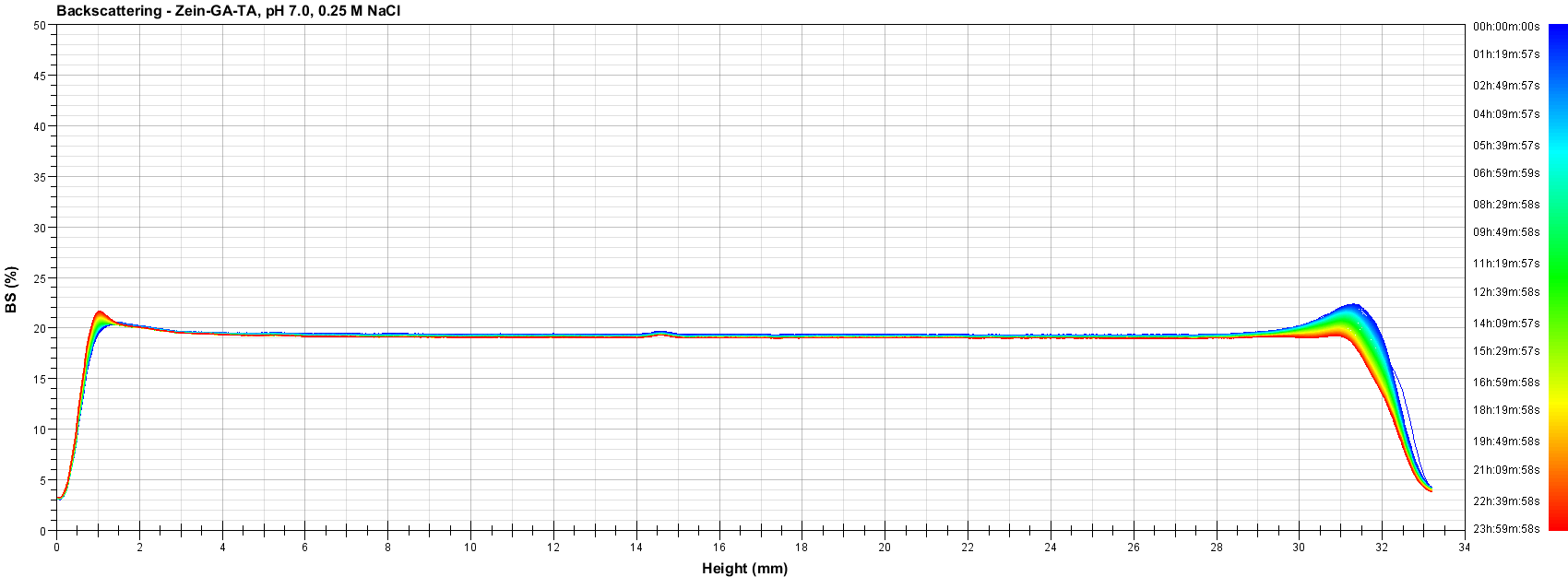


**zein-GA-TA**

**pH 7.0, 0.5 mol/L NaCl**


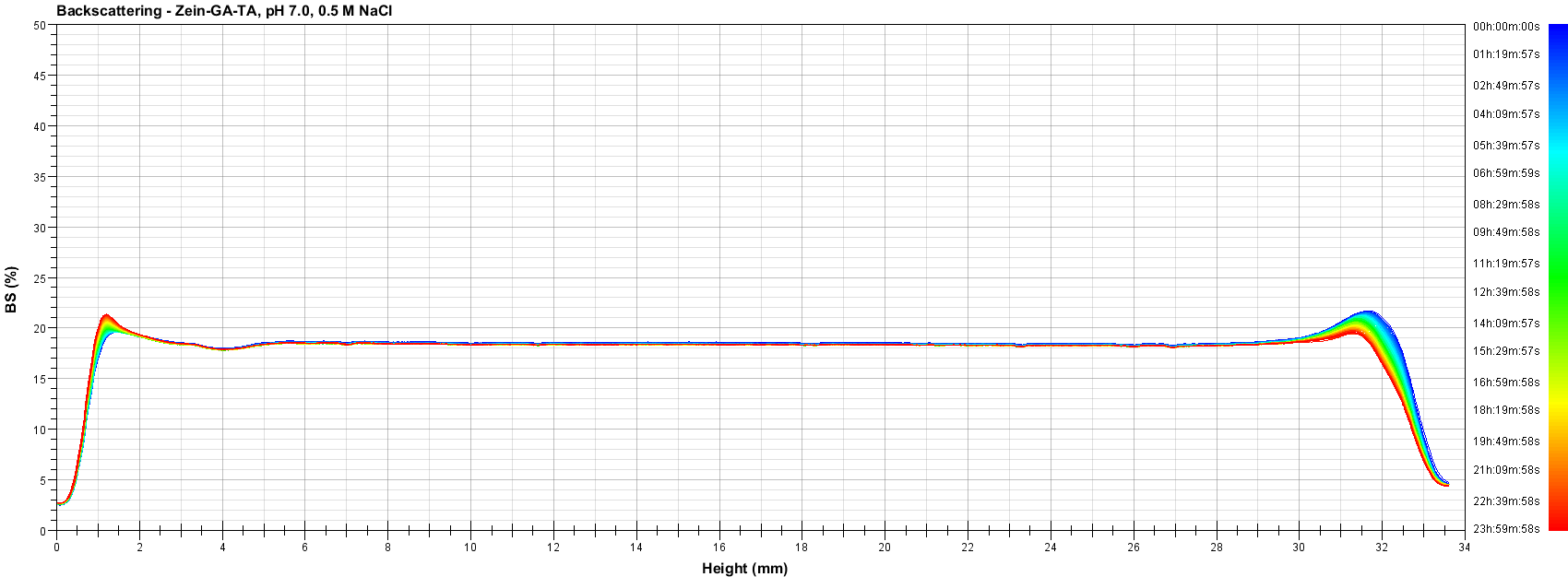


**zein-GA-TA**

**pH 7.0, 1 mol/L NaCl**


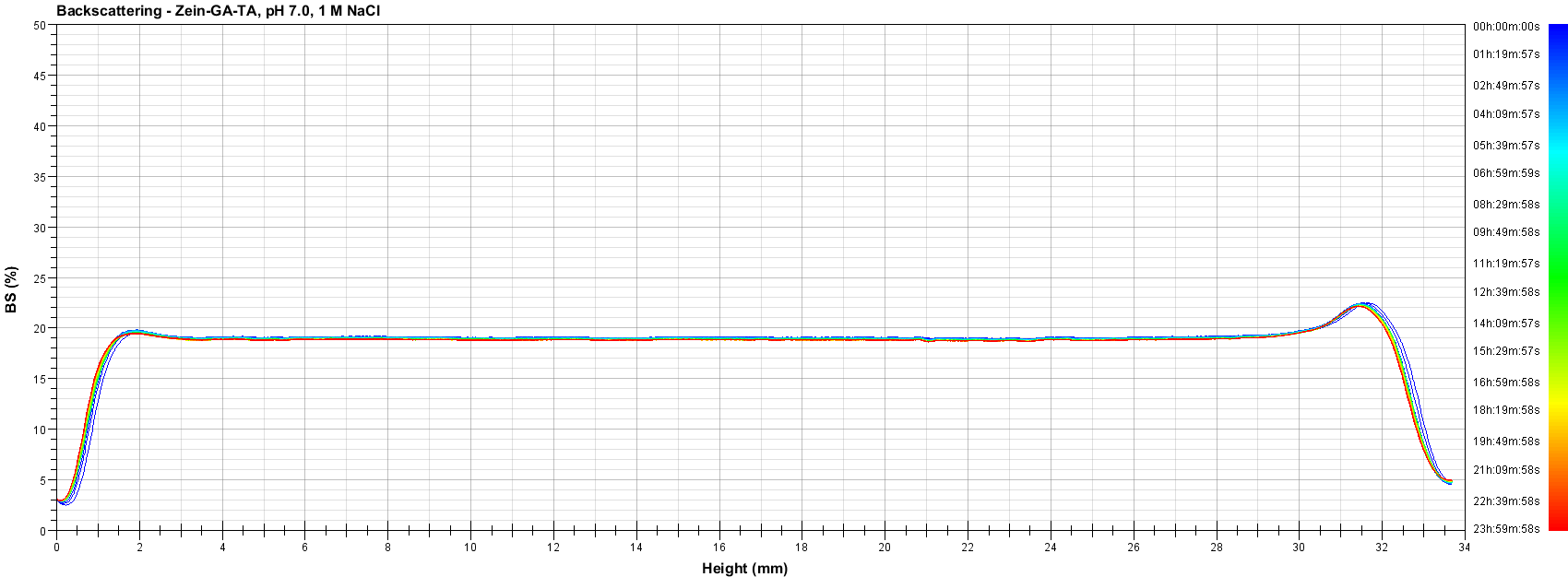


**zein-GA-TA**

**pH 7.0, 2 mol/L NaCl**


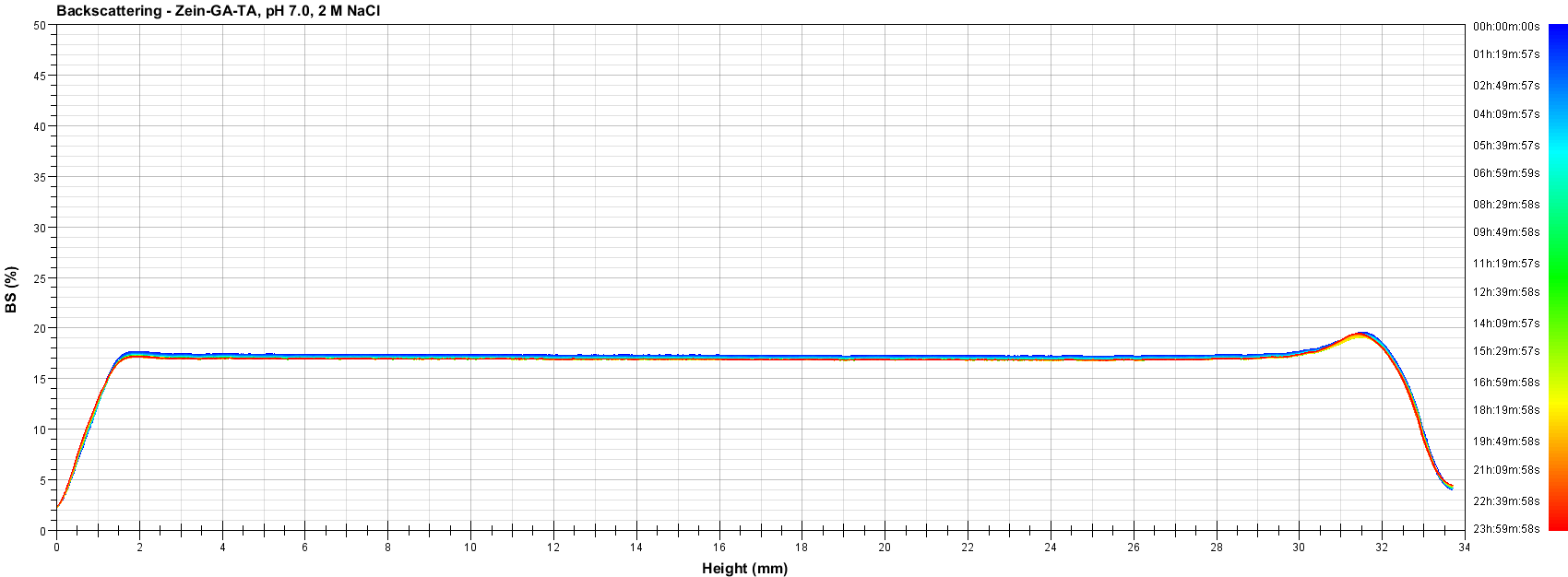


**zein-GA-TA**

**pH 7.0, 3 mol/L NaCl**


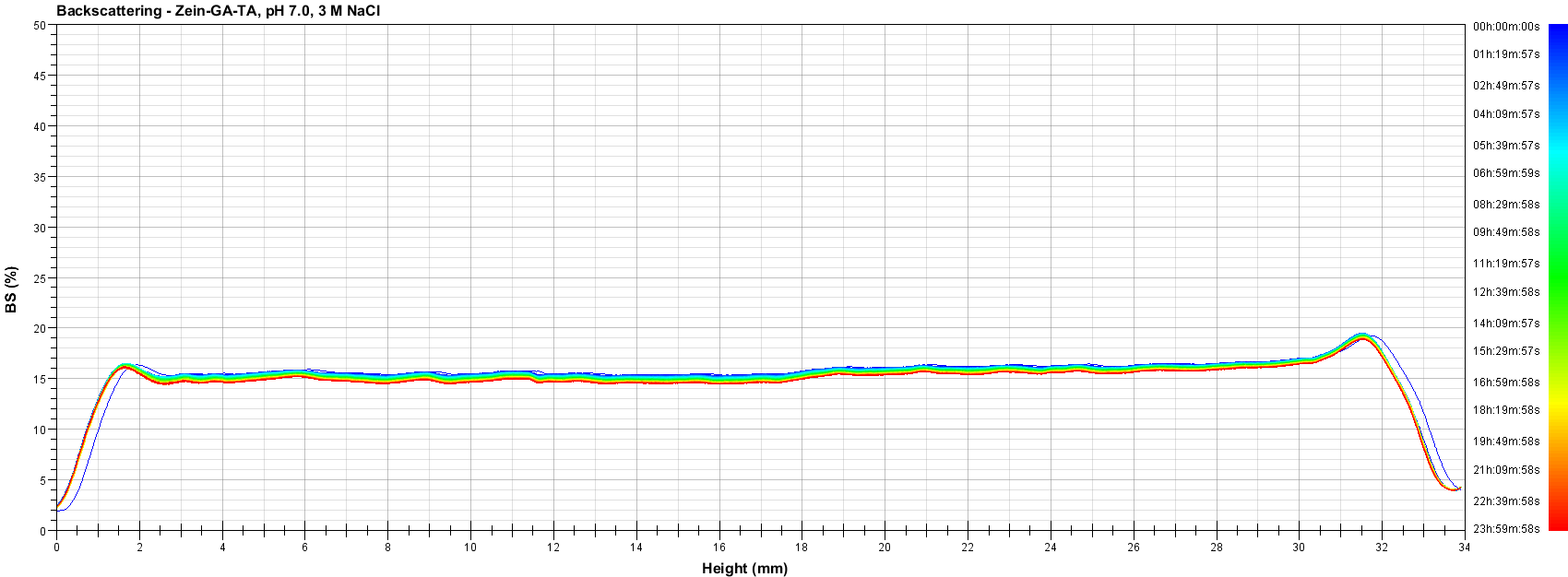


**zein-GA-TA**

**pH 8.5, 0 mol/L NaCl**

**
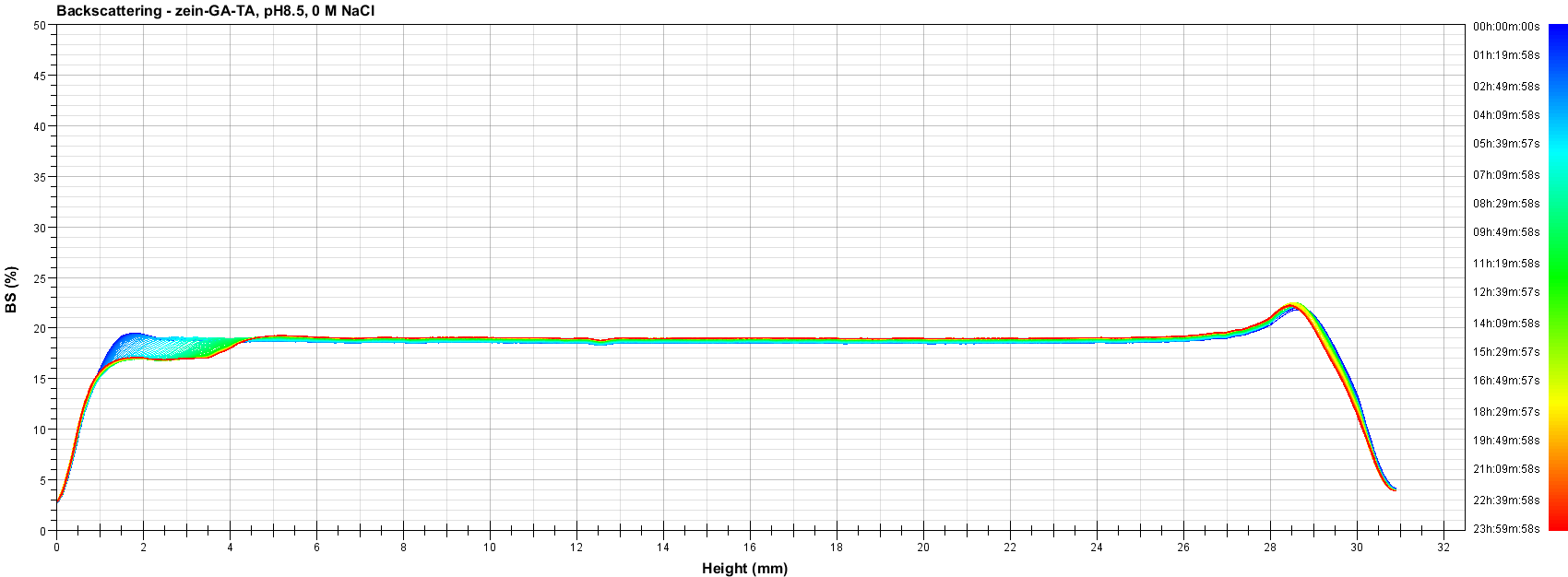
**

**zein-GA-TA**

**pH 8.5, 0.25 mol/L NaCl**

**
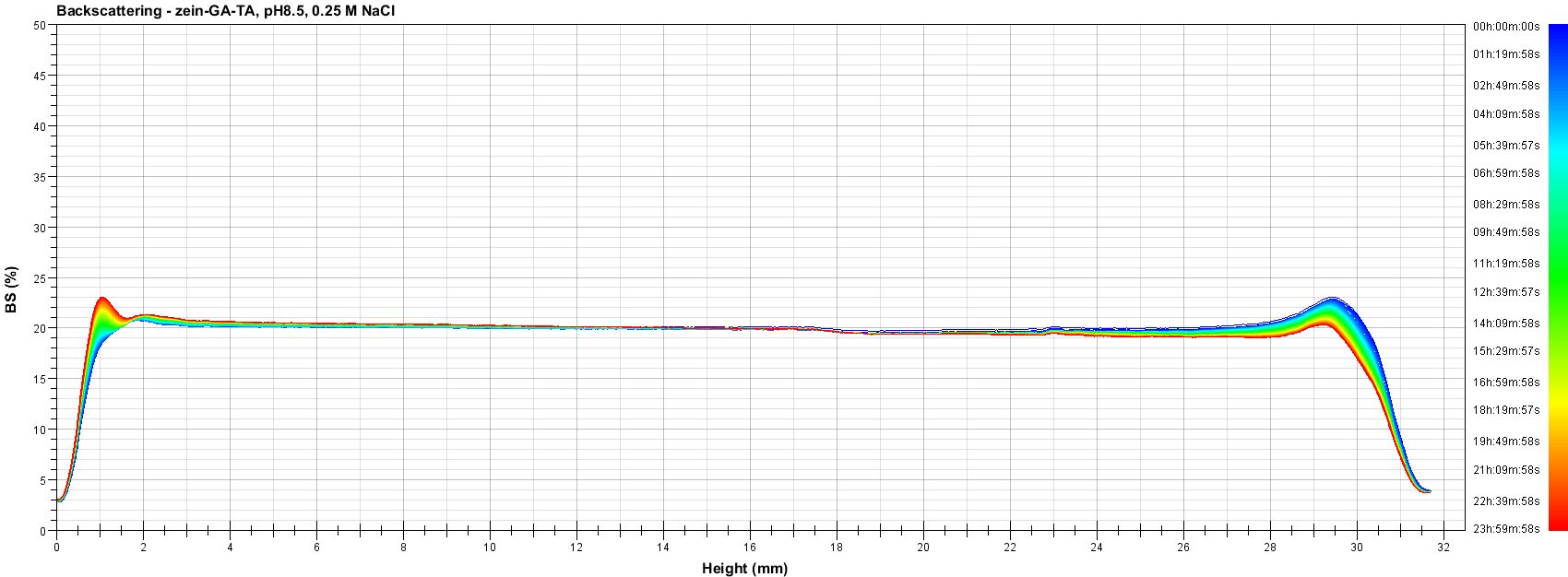
**

**zein-GA-TA**

**pH 8.5, 0.5 mol/L NaCl**


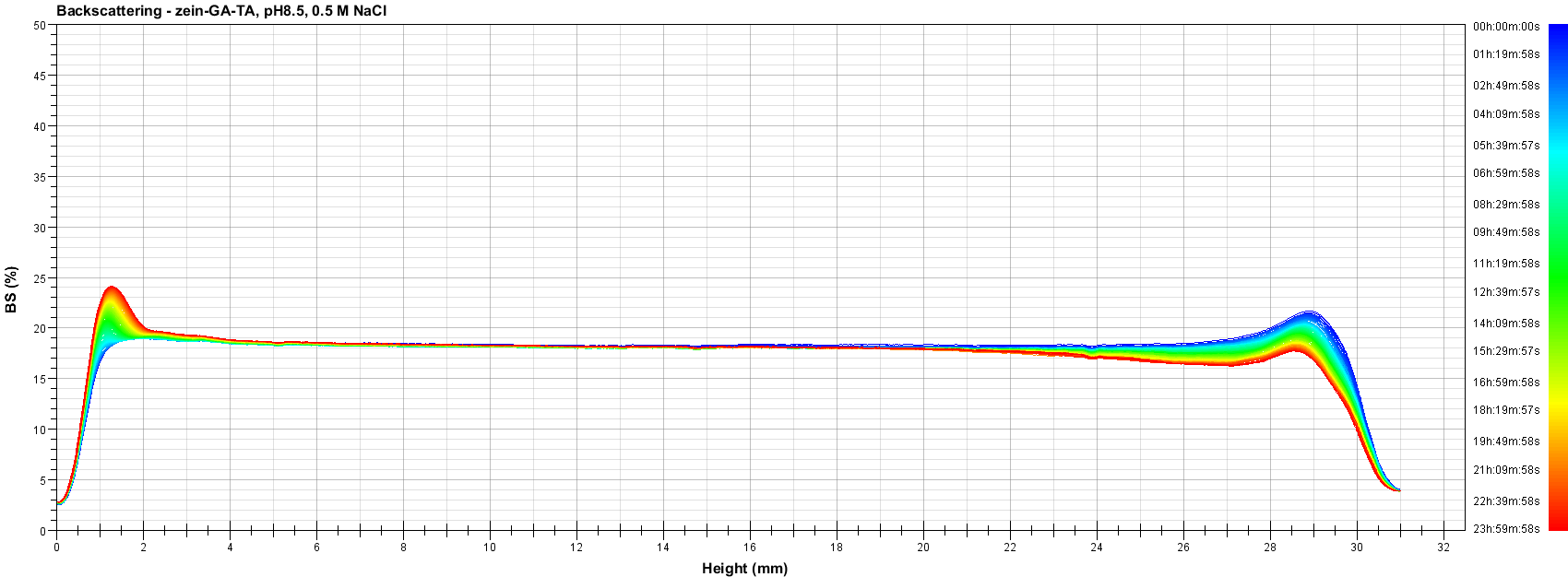


**zein-GA-TA**

**pH 8.5, 1 mol/L NaCl**


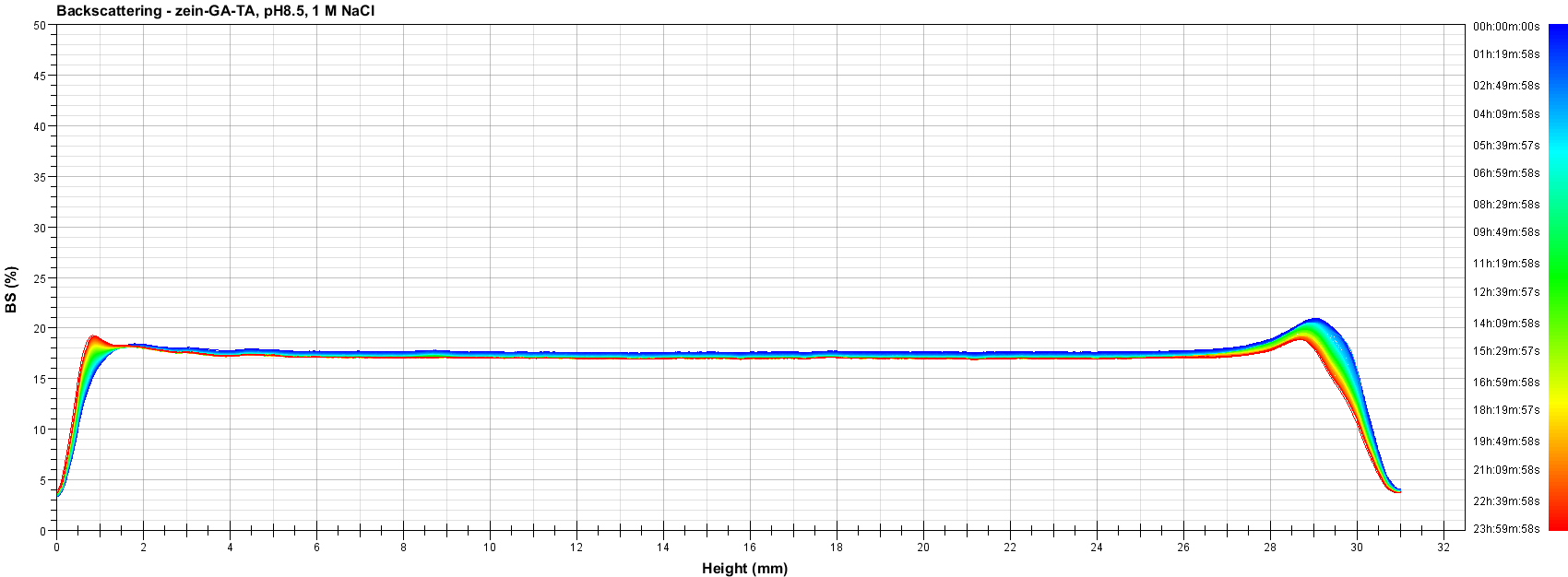


**zein-GA-TA**

**pH 8.5, 2 mol/L NaCl**


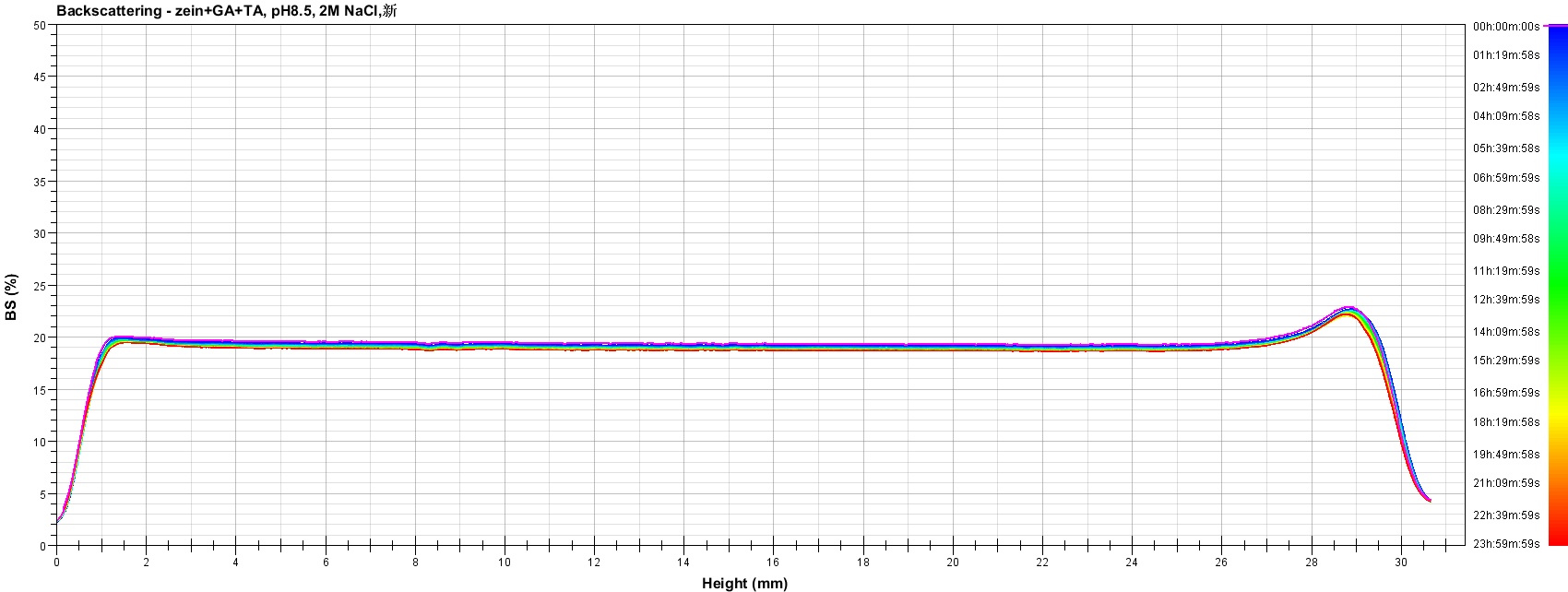


**zein-GA-TA**

**pH 8.5, 3 mol/L NaCl**

**
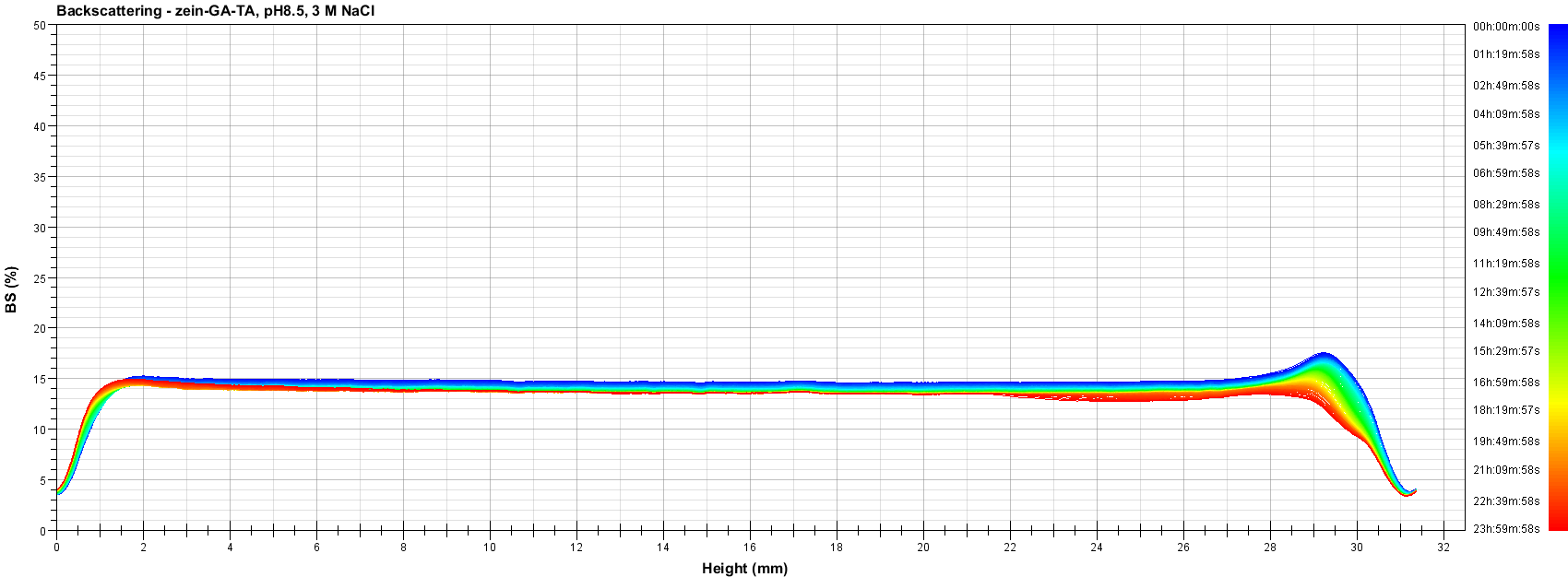
**

**zein-GA-TA**
